# Vermikom feed additive effects on dairy cows’ blood and milk parameters

**DOI:** 10.14202/vetworld.2022.1228-1236

**Published:** 2022-05-21

**Authors:** S. K. Sherimova, N. B. Sarsembayeva, T. B. Abdigaliyeva, B. Lozowicka

**Affiliations:** 1 Department of Veterinary Sanitary Examination and Hygiene, Faculty of Veterinary Science, Kazakh National Agrarian Research University, Almaty, Kazakhstan; 2 Department of Food Biotechnology, Faculty of Food Technologies, Almaty Technological University, Almaty, Kazakhstan; 3 Institute of Plant Protection, National Research Institute, Bialystok, Poland

**Keywords:** diet, feed additive, hematology, milk productivity, milk quality, vermiculite

## Abstract

**Background and Aim:**

Dairy cattle breeding plays a significant role in providing the population with high-quality, reasonably priced goods. The development of this industry and its effectiveness depends on the proper use of available feed products. Feed additives (FAs), as a rule, should compensate for missing elements in the diet. This study aimed to determine the effect of the FA Vermikom on blood parameters, as well as milk physicochemical and mineral composition and yield in lactating dairy cows.

**Materials and Methods:**

A total of 30 Holstein cows, with an average weight of 650±5 kg, were randomly divided into three groups of 10. Over a period of 5 months, each group was fed one of three diets: Mixed main diet without supplements (control), main diet supplemented with 2% Vermikom, and main diet supplemented with 4% Vermikom.

**Results:**

Hematological parameters, productivity, and physicochemical and mineral composition of milk from animals provided the FA Vermikom were higher than those of the control group. In the Vermikom groups, the hemoglobin content was higher by 5.75%, calcium by an average of 10.8%, and total protein by 2.5%. The average daily milk yield was also higher by 8.4% than the control group. Regarding the mineral composition of the milk, the content of calcium, phosphorus, and iron exceeded that of the control on average by 2.9%, 3.4%, and 14.8%, respectively.

**Conclusion:**

We propose introducing 4% Vermikom into the diet of dairy cows based on the study results. Future research will expand our knowledge regarding cows’ needs for all recommended nutrients, thereby improving animal productivity and milk quality. The results obtained contribute to further expanding the food base of animal husbandry in the Republic of Kazakhstan.

## Introduction

An urgent problem of agricultural production in Kazakhstan is the constant and widespread introduction of new technologies to ensure food security. To this extent, high animal husbandry productivity, which includes obtaining the maximum number of food products that meet the requirements of world standards, should be considered [1]. The productivity of dairy cattle largely depends on the completeness of their diet. To organize the full-fledged balanced nutrition of animals, the feed base of farms must be strengthened by procuring high-quality feed additives (FAs) in the required volume and assortment [2]. Cattle diets often lack vital mineral elements [3]. To offset the missing minerals, FAs based on natural aluminosilicates with high sorption properties capable of binding toxins and other chemicals such as zeolite, sapropel, phosphogypsum, and vermiculite, are recommended [4].

One of the most promising natural minerals suitable for use in agriculture is vermiculite [5]. This natural mineral is a product of hydrothermal decomposition of biotite, phlogopite, some chlorites, and other silicates rich in magnesium [6,7]. In the production of vermiculite, high heat treatment leads to an increase in volume and permeability and a decrease in weight. The resulting product is very light and sterile [8] magnesium, aluminum, and iron silicate consisting of silicon dioxide (SiO_2_ ~35-45%), magnesium oxide (MgO ~20-40%), aluminum oxide (Al_2_O_3_ ~7-15%), and iron (III) oxide (Fe_2_O_3_ ~10%) [9]. The material has a relatively high moisture-retaining capacity (200-325% by weight and 20-50% by volume) and thermal conductivity (0.065-0.062 W) with a golden color and a wavy surface [10,11].

The chemical formula of vermiculite depends on the mineral deposit [12]. Elements such as potassium (K), sodium (Na), calcium (Ca), titanium (Ti), and chromium (Cr) may be present in small amounts. Vermiculite subjected to heat treatment can be used with significant economic benefit for various purposes. It does not burn or disintegrate and is chemically inert and biostable. It is also environmentally friendly since vermiculite does not contain impurities that are carcinogenic or harmful to human and animal health [13]. Its natural origin and ability to prolong the effect of the complex trace element preparations enable a reduction in the number of substances used for disease prevention and treatment in animals [14].

The presence of macro- and micro-elements in the composition of vermiculite in a sufficiently large amount distinguishes it from other natural minerals [15]. With vermiculite, animals receive the necessary micro- and macro-elements, and endo- and exo-toxins are excreted from the body, preparing the digestive tract for better assimilation of the essential substances [16]. Vermiculite improves digestive processes by increasing the area of biochemical reactions in the intestine and the sorption of low-molecular-weight metabolites [17].

Large vermiculite deposits have been identified in 40 countries (the United States, Japan, Italy, Canada, Bulgaria, Hungary, etc.). Although vermiculite has been found in many parts of the world, only a few sources have industrial development [18]. In Central Asian countries, large vermiculite deposits have been discovered in Kazakhstan, Kyrgyzstan, and Uzbekistan [19]. Vermiculite is used as a carrier of liquid nutrients because of its high absorbency properties. It is also used as a carrier of vitamins, molasses, choline chloride, and other liquid-based medicinal substances [20]. The production technology, experimental studies, and introduction of biologically active FAs for livestock based on vermiculites are relevant and contribute to the sustainable development of the agro-industrial sector [21].

The aim of the study was to evaluate the effect of the FA Vermikom at different ratios on the general physiological condition of cattle, as well as the impact on milk productivity and mineral composition.

## Materials and Methods

### Ethical approval

The study was approved by the Bioethics Commission of the Kazakh National Agrarian Research University (dated March 15, 2021 No. 148/3) and the analyses comply with the Code of Professional Ethics of Veterinarians, ethical principles of animal research established by the European Convention for the Protection of Vertebrate Animals used for Experimental and other Scientific Purposes.

### Study period and location

The study was conducted from May to October 2021 at the agro-industrial company Kazyna-Zher LTD LLP in the Akdala village of the Turkestan region.

### Animals and animal management

The Vermikom FA comprises 80% expanded vermiculite and 20% sunflower cake. For our study, we obtained Vermikom FA based on expanded vermiculite of the M-150 brand of the Kulantau deposit, a fraction of 5-10 mm. Preliminary results of comparative studies of vermiculite physicochemical and technological properties from various regions of Kazakhstan, Russia, and other foreign countries revealed the optimal parameters for feed preparation to be raw materials of the Kulantau deposit reserves, which amount to more than 3.5 million tons. The economic competitiveness of the Kulantau vermiculite enterprise is due to the proximity of the raw material base and the availability of cheap energy sources, low infrastructure costs, and a convenient transport system [22].

The scientific and economic experiment on cows was conducted using the group method. Animals were distributed into groups according to analog pairs, considering the age, health status, lactation by count, productivity level of the previous lactation, calving and insemination time, live weight, average daily milk yield, and milk fat content (Table-1).

**Table 1:** Live weight and milk production of cows selected for the study (average values).

Indicators	Groups		

	Control	2% FA group	4% FA group
Number of heads	10	10	10
Live weight, kg	652±2.3	650±6.7	653±5.3
Average daily milk yield, kg	18.3±1.23	18.9±0.08	18.2±1.14
Fat content in milk, %	3.01±0.53	3.12±0.41	3.08±0.05
Duration of lactation	5	3	5
Period between calvings, days	350±4.7	348±3.4	351±5.32
Health status	Healthy	Healthy	Healthy

No significant differences in quantitative indicators were observed between groups. The reliability of the obtained data was assessed using the method of variation statistics based on Student’s t-test

Three groups of ten lactating Holstein cows with an average weight of 650±5 kg were evaluated from the beginning of the lactation period (three to five lactations) over 152 days according to the scheme presented in Table-2. During the experiment, the conditions of maintenance and care for all groups were the same. The cows were milked 3 times a day. In the preliminary period (10 days), cows were selected, and the experimental groups were formed. During a transitional period (7 days), the animals were acclimatized to eating the diets under evaluation. During the main period of the experiment (135 days), lactating cows of the control group received a mixed main diet without any supplement, whereas the experimental groups received the main diet supplemented with 2% or 4% Vermikom FA. In the final period (2 days) of the experiment, all animals were transferred to the main diet without the FA.

**Table 2:** Scheme of the experiment.

Experimental period	Cow group	Number of heads	Duration, days	Feeding features
Preliminary	Control	10	10	MD
	2% FA group	10		MD+2% Vermikom FA
	4% FA group	10		MD+4% Vermikom FA
Transitional	Control	10	7	MD
	2% FA group	10		MD+2% Vermikom FA
	4% FA group	10		MD+4% Vermikom FA
Main	Control	10	135	MD
	2% FA group	10		MD+2% Vermikom FA
	4% FA group	10		MD+4% Vermikom FA

MD=Main diet, FA=Feed additive.

The diets were composed considering the cows’ age, physiological state, live weight, milk productivity, keeping conditions, fatness, and time since the beginning of lactation and balanced based on the chemical analyses of feed for normalized nutrients according to the detailed norms of the farm in question considering the production of 20-21 L of milk with a fat content of 3.8-4.0%/head/day.

According to the composition and quantity of feed, the diets of all groups were the same, differing only in the absence or presence of Vermikom. The daily diet for all groups on average for the main period of the experiment included 30.0 kg of corn silage, 6.02 kg of a mixture of concentrates, 3.40 kg of legume hay, 2.50 kg of grain hay, and 1.45 kg of feed molasses. The use of FAs under evaluation affected feed consumption. The control and 2% FA group cows completely consumed concentrated feed, hay, and molasses. Silage consumption was 96.5% in the control group and 98.2% in the 2% FA group. The cows in the 4% FA group consumed the specified feed completely.

Indicators such as temperature, humidity, and air velocity in the rooms where cows were kept were studied over time according to generally accepted hygienic methods. The concentrations of carbon dioxide and ammonia were also determined with a UG-2 gas analyzer. Fluctuations in indoor air temperature and humidity were recorded by weekly M-16A thermographs (Zapadpribor LLC, Russia) and weekly M-21AN hygrographs (Prompribor CJSC, Yekaterinburg, Russia). A spherical katathermometer measured the air velocity in the rooms. The ammonia and carbon dioxide content was measured with a gas analyzer UG-2 (Technogaz LLC, Belgorod, Russia). All indoor microclimate indicators were determined for 3 consecutive days a month, 3 times a day, in the morning, afternoon, and evening at three points along the diagonal of the room on two levels at heights of 30-100 cm (in the area where animals were located) and 1.5 m (in the area where humans were located).

### Sampling and hematological examination of cows’ blood

The state of natural resistance and morphological and biochemical parameters of the blood were studied before (in the first week of May 2021), during (in the third week of July 2021), and before completion of the experiment (in the first week of October 2021). Blood samples (8-10 ml) were taken from the jugular vein before morning feeding from five animals per group in compliance with the rules of asepsis and antiseptics. The blood for analysis was taken in a clean test tube with 10% EDTA solution to determine erythrocytes, hemoglobin, and leukocytes. Another test tube was used to collect blood for serum. Serum samples were separated by centrifugation at 604× g for 10 min in a refrigerated laboratory centrifuge (LMC-4200R [Biosan, Latvia]). Before taking blood from cows, the timing of vaccinations and other veterinary and health measures were considered.

Other equipment utilized for determining blood parameters included a thermostatic bath LAB-TJ-TB-01/19 (LOIP CJSC, St. Petersburg, Russia), electronic laboratory scales model CE 224-C (Mir Vesov LLC, Moscow, Russia), a SAMSUNG refrigerator, measuring flasks, biological test tubes, Florinsky PFX-1-14×60 tubes, a one-channel pipette dispenser ECOHIM-OP-1-5-50, and a one-channel pipette dispenser Color DPOPTS-1-20-200.

Hemoglobin, leukocyte, and erythrocyte values were assessed using an automatic hematological analyzer for veterinary medicine VS-2900 Vet Plus (Mindray, China). The serum carotene content was determined using the photometric method. The total protein in the serum was calculated by the refractometric method using an IRF-454 B2M refractometer (NV-Lab Kazakhstan LLP, Almaty, Kazakhstan). The inorganic phosphorus content was determined with a Biochem Sa medical laboratory (High Technology, Inc., United States) photometer. The glucose oxidase method (AGAT-MED LLC, Moscow, Russia) was used to determine glucose in biological fluids. Total calcium in serum was analyzed using the Wilkinson complexometric method, and the diffuse method was used to determine the alkaline reserve in the plasma.

### Investigation of the physicochemical and mineral composition of milk

Milk sampling from flasks was taken by immersing a clean, dry tube at such a speed that milk entered it simultaneously [23]. After closing the upper hole tightly with the thumb, the tube was quickly removed, and the milk was poured into a clean, dry bottle with a rubber stopper. Bottles with milk samples were labeled with appropriate inscriptions and stored in the refrigerator at a temperature of 4°C until the next day. The volume of each milk sample was 300-500 mL.

To determine the amount of fat, protein, and milk density, a Lactane 600 analyzer (Russkaya Ferma LLC, Russia) was used. The pH value and titrated acidity were determined using a titrator TitroLine 5000 (SI Analytics, Germany). Physicochemical and analytical studies of milk were conducted in the Food Safety Research Institute laboratory. Milk productivity was measured during lactation by a control milking every 10 days. The mass fraction of protein, fat, and density was determined in the average milk sample from each cow. The mineral composition of milk was determined using the KVANT.Z atomic absorption spectrometer (KORTEK LLC, Moscow, Russia).

### Statistical analysis

The data obtained were subjected to variational and statistical processing using Student’s t-test on a personal computer with Microsoft Excel. The differences were considered statistically significant at p<0.05.

## Results

### Blood parameters

Changes in the hematological status of the cows under the influence of Vermikom FA were evaluated (Table-3).

**Table 3:** Dynamics of hematological parameters of cows’ blood (X±Sx).

Indicator	Experimental period	Group			Normal

		Control	Experimental		

			2% FA group	4% FA group	
Hemoglobin, g/L	At the beginning	107.23±1.01	106.34±1.23	107.51±1.31	108-115
	At the end	108.51±0.11	109.21±1.12*	115.14±0.02*	
Erythrocytes,^10-12^/L	At the beginning	5.08±0.08	5.09±0.07	5.45±0.13	5-10
	At the end	5.14±1.25	5.53±2.31	5.64±0.05	
Leukocytes, 10^9^/L	At the beginning	7.16±0.14	7.15±0.22	7.46±0.23	4-12
	At the end	9.51±0.01	8.33±0.23*	8.51±2.31	
Alkali reserve, voL/% CO_2_	At the beginning	27.24±0.81	27.14±0.55	27.41±0.64	30-46
	At the end	29.21±0.65	28.56±0.02*	29.84±0.05	
Carotene, mg/%	At the beginning	0.46±0.72	0.45±0.89	0.46±0.64	0.4-1.0
	At the end	0.44±0.02	0.44±0.05	0.48±0.31	
Total calcium, mg/%	At the beginning	9.36±0.16	9.29±0.19	9.44±0.14	10-12.5
	At the end	9.42±0.01	10.25±0.15	10.89±0.54*	
Inorganic phosphorus, mg/%	At the beginning	5.79±0.03	5.80±0.06	5.68±0.06	5.8-7.8
	At the end	5.91±0.01	6.65±0.32*	7.12±0.25*	
Total protein, g/L	At the beginning	72.38±1.54	71.21±0.98	72.39±1.42	62-82
	At the end	72.42±0.06	73.08±0.52*	72.56±1.42	

* p<0.05

At the beginning of the study, the hemoglobin content in all groups was not significantly different and ranged from 106.34 to 107.51 g/L. At the end of the study, the hemoglobin of cows receiving 2% FA was 109.21±1.12 g/L, and those in the 4% FA group reached 112.14±0.02 g/L, which was 5.75% higher than that of the control group (p<0.05). At the beginning of the experiment, erythrocytes averaged 5.11±0.42×10^12^/L for all cows. At the end of the study, erythrocytes in the control group were 5.14±1.25×10^12^/L, whereas cows that received 4% FA had a red blood cell content of 5.64±0.05×10^12^/L, which was 8.9% more than the control group. The number of erythrocytes in cows fed 2% FA was 5.53±2.31×10^12^/l at the end of the study, which was 2% less than the 4% FA group (p<0.05).

By the end of the experiment, the number of leukocytes in the blood of cows in the control group slowly increased from 7.16±0.14×10^9^/L to 9.51±0.01×10^9^/L. In the 2% FA group leukocytes increased from 7.15±0.22×10^9^/L to 8.33±0.23×10^9^/L, and in the 4% FA group from 7.46±0.23×10^9^/L to 8.51±2.31×10^9^/L. This indicator was lower by 1.3±1.08×10^9^/L in the control group and 1.1±0.06×10^9^/L in the 2% FA group (p<0.05).

At the beginning of the experiment, the total calcium content in all groups was 9.29-9.44 mg/%. In the cows that received Vermikom FA, the calcium content increased. The calcium content of the control group cows did not change and was correspondingly lower by 8.1% compared with the 2% FA group and 13.5% compared with the 4% FA group. Likely, the amount of calcium in the blood of the control group indicates the use of this element for the growth and development of the fetus, showing insufficient intake of calcium with standard feed.

The same can be noted concerning the content of inorganic phosphorus, which had not changed significantly in the serum of cows of the control group. Moreover, the amount of inorganic phosphorus in the blood of cows of the experimental groups, compared with the control group, was higher by 11% in the 2% FA group and 16% in the 4% FA group (p<0.05).

In dynamics, the level of alkali reserve in the blood of all cows changed slightly. At the beginning of the experiment, the level of alkali reserve in cows of the 2% FA group was 27.14±0.55 voL/% and 27.41±0.64 voL/% in the 4% FA group. At the end of the study, the level of alkali reserve in the blood of cows of the 2% FA group had reached 28.56±0.02 voL/%, which was 2.2% lower than the control group. In cows of the 4% FA group, the level of alkali reserve at the end of the experiment was 29.54±0.05 voL/%, which was 2.1% higher than the control group.

At the beginning of the experiment, the total protein content in the serum of the control and experimental groups ranged from 71.21±0.98 to 72.39±1.42 g/L. When 2% FA was included in the diet, the total protein was 73.08±0.52 g/L. When 4% FA was included in the diet, the total protein was 73.08±0.52 g/L, which was 2.5% higher than at the beginning of the experiment in this group (p<0.05).

The amount of carotene in the blood of cows of the control group was constantly decreasing, apparently due to the content of carotene in feed and the physiological state of the cows’ bodies. Before the experiment, the amount of carotene in the serum of the control group was 0.46±0.72 mg/%, whereas it equaled 0.45±0.89 mg/% in the 2% FA group. In the 4% FA group, by the end of the experiment, carotene was 0.48±0.31 mg/%. In cows fed 2% FA, carotene was 8.3% less than in the 4% FA group. The inclusion of a FA based on vermiculite in the cows’ diet contributed to a favorable effect on their hematological picture. According to our results, the inclusion of vermiculite at 4% of feed dry weight positively affected blood composition (p<0.05).

### Milk productivity and physicochemical properties

We assessed milk productivity as well as the physicochemical properties of the milk (Table-4). The use of the Vermikom FA in the cows’ diets positively affected milk yield and quality. The animals that received 4% FA had significant superiority in average daily milk yield compared with the control and 2% FA groups. The addition of 4% FA positively affected the average daily milk yield of 24.81±0.11 l (p<0.05). Based on the average daily milk yield, cows in the 2% FA group exceeded the control group by 2.65%, and cows in the 4% FA group had an average daily yield of 14.2% more with a statistically significant difference of p<0.05. Note that the difference between the animals of the 2% FA and 4% FA groups was 11.8%.

**Table 4:** Indicators of milk productivity and physicochemical properties of milk at the end of the experiment (M±m).

Indicator	Group		

	Control	Experimental	

		2% FA group	4% FA group
Average daily milk yield, L	21.28±0.31	21.86±0.32	24.81±0.11*
Mass fraction of fat, %	3.21±0.06	3.25±0.05*	3.45±0.03*
Mass fraction of protein, %	2.96±0.03	2.98±0.01	3.02±0.03*
Density, kg/m^3^	1,028.1±0.03	1,028.1±0.01	1,028.3±0.1
Acidity, T	17.2±0.01	17.1±0.02	17.2±0.01

* p<0.05

During the research period, the animals of the 4% FA group had a higher fat content in milk, that is, the fat content of the 4% FA group increased by 6.9% compared to the control group (p<0.05). The fat content was almost the same in the control group (3.21±0.06%) and the 2% FA group (3.25±0.05%) (p<0.05). In the 4% FA group, the fat content in milk was 5.7% higher than in the 2% FA group.

Cows that received FA also had higher milk protein content. The 2% FA group had an advantage over the cows from the control group by 0.6% and 1.9% from the 4% FA group (p<0.05). The density and acidity of the milk did not show a significant difference, which suggests that feeding Vermikom did not significantly affect those physicochemical indicators. Nonetheless, the use of Vermikom in the diet of dairy cows contributed to increased milk productivity and quality.

### Evaluation of the effect of Vermikom FA on the mineral composition of milk

The introduction of Vermikom FA into the diet of dairy cows had a positive effect on the total mineral content of their milk (Table-5). On average, during the entire study period, the milk of the 4% FA group was superior to that of animals in control and 2% FA groups.

**Table 5:** Mineral content in cows’ milk, (M±m, n=10).

Element	Experimental period	Group		

		Control	Experimental	

			2% FA group	4% FA group
Ca, mg/%	At the beginning	65.4±4.14	64.4±2.11	65.3±3.52
	At the end	120.1±2.31	121.2±1.16	126.3±3.81*
	On average	92.7±3.22	92.8±1.63	95.8±3.66
P, mg/%	At the beginning	60.5±3.72	60.8±2.23	59.5±0.12
	At the end	71.8±3.21	75.5±0.52*	78.2±2.41*
	On average	66.15±3.46	68.15±1.37	68.85±1.26
Mg, mg/%	At the beginning	14.5±0.91	14.4±0.32	14.5±0.41
	At the end	15.6±0.81	15.8±0.52	16.6±0.01*
	On average	15.1±0.86	15.1±0.42	15.5±0.21
Fe, mg/kg	At the beginning	0.18±0.004	0.19±0.023	0.18±0.001
	At the end	0.22±0.021	0.24±0.041	0.28±0.004*
	On average	0.20±0.012	0.21±0.032	0.23±0.002
Cu, mg/kg	At the beginning	0.020±0.002	0.021±0.002	0.020±0.003
	At the end	0.018±0.004	0.019±0.001	0.021±0.004
	On average	0.019±0.003	0.020±0.001	0.020±0.003
Zn, mg/kg	At the beginning	1.54±0.001	1.53±0.052	1.53±0.002
	At the end	1.57±0.003	1.59±0.003*	1.65±0.021
	On average	1.55±0.002	1.56±0.027	1.59±0.011
Mn, mg/kg	At the beginning	0.017±0.001	0.017±0.002	0.016±0.014
	At the end	0.019±0.052	0.020±0.021	0.021±0.002*
	On average	0.018±0.026	0.018±0.011	0.018±0.008

* p<0.05. Ca=Calcium, P=Phosphorus, Mg=Magnesium, Fe=Iron, Cu=Copper, Zn=Zinc, Mn=Manganese

Analyzing the calcium content in cows’ milk at the end of the lactation experiment, we found an increase in the calcium content in the 2% and 4% FA groups by 0.9% and 4.9%, respectively, in comparison with the control group. During the period when 4% Vermikom FA was used, the highest calcium level was observed, amounting to 126.3±3.81 mg/% (p<0.05). At the beginning of the experiment, the calcium content of all groups was similar, ranging from 64.4 to 64.5 mg/%. The cows of the 2% FA group had a decrease in milk calcium content compared with those of the 4% FA group by 4.03%. On average, over the entire study period, an increase in calcium content was observed in the milk of all groups (92.7±3.22 mg/% in controls, 92.8±1.63 mg/% with 2% FA, and 95.8±3.66 mg/% with 4% FA).

The phosphorus content of all study groups at the beginning of the experiment was almost the same at 60.5±3.72 mg/% in the control group, 60.8±2.23 mg/% in the 2% FA group, and 59.5±0.12 mg/% in the 4% FA group. At the end of the experiment, the phosphorus content was highest (78.2±2.41 mg/%) in the milk of the 4% FA group, outperforming that of the control and 2% FA groups by 8.2% and 3.4%, respectively (p<0.05). In addition, compared with the control group, the phosphorus index at the end of the experiment was 4.9% higher at 75.5±0.52 mg/%. The average lowest phosphorus content was observed in the control group at 66.15±3.46 mg/%. Considering this indicator for the entire period of the experiment, the highest value was found in the 4% FA group (68.85±1.26 mg/%) (p<0.05), which was 3.9% and 1.01% more than that of cows in control and 2% FA groups, respectively. The average phosphorus content in the milk of cows of the 4% FA group (68.15±1.37 mg/%) increased by 2.9% compared with the control group and decreased by 1.01% compared with the second experimental group. The ratio of calcium and phosphorus is of great importance when assessing the biological usefulness of milk. The ratio of 1.5:1 is considered optimal. In our studies, this indicator was 92.7 and 66.1 in the control group (mg/%), 92.8 and 68.1 in the 2% FA group (mg/%), 95.8 and 68.8 in the 4% FA group (mg/%).

Regarding the magnesium content in milk, an increase in all groups was noted for the entire study period. The average value of magnesium was 15.1±0.86 mg/% in the control group, 15.1±0.42 mg/% in the 2% FA group, and 15.5±0.21 mg/% in the 4% FA group (p<0.05). As for the milk iron content, at the end of the study, we observed an increase in all groups, 18.1% in the control group, 20.8% in the 2% FA group, and 35.7% in the 4% FA group (p<0.05). The maximum indicator was noted in cows’ milk of the 4% FA group at 0.28±0.004 mg/kg, which was 21.4% and 14.2% more than the control and 2% FA groups, respectively (p<0.05). The lowest iron content was noted in the control group at 0.22±0.021 mg/kg, which was 8.3% less than the 2% FA group (p<0.001). During the entire period of research, the average iron content detected in the 4% FA group was 0.23±0.002 mg/kg. The average iron content in cows’ milk of 2% FA group was 0.21±0.032 mg/kg. The control group had the minimum average milk iron content at 0.20±0.012 mg/kg.

At the beginning of the experiment, the minimum copper content in milk was observed in the control group (0.020±0.002 mg/kg) and the 4% FA group (0.020±0.003 mg/kg), which was 4% less than that of the 2% FA group. At the end of the experiment, the superiority of the 4% FA group was observed for this indicator at 0.021±0.004 mg/kg, which was more than 14.2% of the control group and 9.5% of the 2% FA group. The lowest average copper content was noted in the milk of the control group at 0.019±0.003 mg/kg. For the entire study period, the highest copper value was found in the FA groups (within 0.020 mg/kg), which was 5% more than the control group.

At the end of the experiment, the highest zinc content was detected in the milk of the 4% FA group at 1.65±0.021 mg/kg. By the end of the experiment, there was a 1.9% increase in the zinc content in the milk of the control group; however, it was lower than the FA groups by 1.2% for the 2% group and 4.8% for the 4% FA group. The maximum average was found in cows’ milk of the 4% FA group at 1.59±0.011 mg/kg (p<0.05). The lowest average zinc value for the entire period was observed in the control group at 1.55±0.002 mg/kg.

The dynamics of the manganese content in milk at the beginning of the experiment were within the limits of 0.017±0.008 mg/kg. At the end of the study, the maximum indicator was noted in the milk of cows of the 4% FA group at 0.021±0.002 mg/kg. The minimum manganese content was found in the control group at 0.019±0.052 mg/kg. Over the entire experiment period, the average content of manganese in the milk of the control and experimental groups was 0.018±0.022 mg/kg (p<0.05).

## Discussion

To strengthen the dairy cattle industry and reduce dependence on imports, it is necessary to improve animal nutrition using the highly effective method of balancing diets. This technique will reduce the consumption of basic feed and the cost of production and consequently increase profitability [24,25]. The use of Vermikom FA had a significant impact on the morphological blood parameters of cows. The morphological composition of the blood changed the quantitative composition of the elements. During the experiment, an increase in erythrocytes and hemoglobin was noted in the Holstein cows consuming the supplement, which is a positive factor indicating a high level of metabolic processes. The erythrocytes in the blood of pregnant cows receiving the FA increased by 8-9%, and the hemoglobin level in the blood increased by 5-6%.

In the work of Consigliere *et al*. [26], the effects of low doses of FAs based on vermiculite on the characteristics of carcasses and the quality of pig meat were investigated. According to the published results, a diet that included a low dose of clay minerals could be useful for improving the characteristics of the carcass and the quality of the pig meat. Nanostructured vermiculite with a particle size from 50.0 to 160.0 nm was used as a FA in fattening bull diets in studies by Smolentsev *et al*. [27]. According to the results, the functional and technological properties of the meat of bulls fed diets enriched with nanostructured vermiculite were of higher quality and were more favorable for further processing. In the studies of Fatkullin *et al*. [28], the effect of Vermiculite FA on the live weight of Kazakh white-headed bulls showed that during the entire period of the experiment, the bulls of the experimental group had the highest indicators of average daily growth compared with their peers in the control group. Since the nature of changes in metabolic processes in the body of animals affects the blood, the determination of its constituent components acquires a particular significance [29].

Similar results to those of our study were reported by Kardaya *et al*. [30], who researched the effects of zeolite in cow diets on mineral and hematological blood parameters. The inclusion of zeolite in cow diets improved the amount of iron and most hematological parameters. The values of the analyzed indicators tended to increase in the blood of cows of all experimental groups by the end of the study. As for leukocytes, the opposite pattern has been established. Optimization of mineral nutrition of cows allowed for the correction of several biochemical parameters without harming their body. All changes in the morphological composition of the blood of cows occurred within the physiological norm set for cattle.

Analysis of the data revealed results consistent with our previous work in which we investigated the effect of expanded vermiculite on the blood composition of laying hens [21]. The number of leukocytes decreased by 10.5-12.4% compared with the control group. Based on the data obtained, it can be concluded that the introduction of FAs positively affected the mineral composition of cows’ milk (p<0.05). The use of Vermikom FA helped improve the mineral composition of the blood. In the cows of the experimental groups, the total calcium in the serum increased to 13.5%. These data correspond to the works of Khachlouf *et al*. [31], who studied the effect of zeolite in the diet of lactating cows on the content of calcium, phosphorous, and magnesium in the blood. The addition of zeolite significantly increased the calcium content in plasma. These results show that natural minerals can be effectively used in the diets of lactating cows with a positive effect on milk production and blood parameters. Santos investigated using natural aluminosilicates to reduce hypocalcemia in dairy cows and reported positive results [32].

The amount of inorganic phosphorus in the blood of cows of the experimental groups where our FA was used was 11-16% higher than the control group. Similar results were obtained by I. Folnožić [33], who studied the effect of natural clinoptilolite on phosphorus, potassium, and sodium levels in the serum of lactating cows during early lactation. Accordingly, the use of Vermikom FA in cows contributed to improvement in the mineral composition of the blood.

One of the main indicators considered when performing scientific and economic experiments on lactating cows is milk productivity [34]. According to the results of our study, it can be concluded that the addition of Vermikom to the cows’ diet had a positive effect on milk yield. Cows who received the 4% FA had the most significant superiority in average daily milk yield compared with the control and 2% FA groups. Khachlouf *et al*. [35] also studied the effect of natural minerals such as zeolite on production indicators and the condition of scars of lactating cows. Zeolite in the diet had a significant effect on dairy products and the characteristics of scars, proving once again that the use of natural aluminosilicates has a positive effect on the productivity of dairy cows.

The positive effect of the FA vermiculite was studied in detail by Consigliere *et al*. [36], who found that the average growth rate of pigs was higher in those that received the FA compared with the control group. Tyurina *et al*. [37] detailed the results of the effect of a mineral mixture with vermiculite on the physiological parameters of broiler chickens, where vermiculite improved the digestibility of feed nutrients.

According to our data, the average daily milk yield of cows of the experimental groups exceeded the control group by 2.65% and 14.2%. According to physicochemical indicators, in the experimental group where 4% FA was used together with the main diet, the milk had a higher fat and protein content. Our data are consistent with the results of Đuričić *et al*. [38], who found that the use of dietary clinoptilolite with pine fodder positively affected the ratio of milk fat and protein during early lactation in Holstein cows.

The introduction of the Vermikom FA into cows’ diet positively affected the mineral composition of milk. The amounts of elements such as calcium, phosphorus, magnesium, iron, and manganese at the end of the experiment in the milk of cows of all experimental groups had increased compared with the control group. In the experimental groups that received minerals in addition to the diet, there was no depletion of the mineral composition of milk, which is important since milk, besides having high nutritional value, is the main raw material for the dairy industry and the mineral composition determines its technological properties. In his research, Osman studied the effect of zeolite in the diet of lactating cows on milk yield and milk composition. The addition of zeolite at 80 g/day significantly increased the content of elements in milk [39]. These results indicate that natural minerals positively affect the physiological state of cows and the mineral composition of milk.

## Conclusion

It can be concluded that the Vermikom FA had a positive effect on the studied indicators, which were within the limits of physiological norms. Our study suggests that the Vermikom FA, which has absorption and ion exchange, molecular force, and catalytic properties, can be used in cow feed at the rate of 4% of the dry matter weight of the diet in production conditions. Additional evaluations are needed in a large sample population to confirm our results.

## Authors’ Contributions

SKS: Acquisition of data, drafting the manuscript. NBS: Conception and design of the study, analysis and interpretation of data, and revised the manuscript. TBA: Conception and design, analysis and interpretation of data, and drafted the manuscript. BL: Critically revised the manuscript. All authors read and approved the final manuscript.
